# Impact of age to ferritin and neutrophil‐lymphocyte ratio as biomarkers for intensive care requirement and mortality risk in COVID‐19 patients in Makassar, Indonesia

**DOI:** 10.14814/phy2.14876

**Published:** 2021-05-27

**Authors:** Haerani Rasyid, Alvin Sangkereng, Tutik Harjianti, Audrey S. Soetjipto

**Affiliations:** ^1^ Department of Internal Medicine Faculty of Medicine Hasanuddin University Makassar Indonesia; ^2^ Department of Internal Medicine Siloam Hospitals Makassar Makassar Indonesia; ^3^ Siloam Hospitals Makassar Makassar Indonesia

**Keywords:** age, COVID‐19, ferritin, neutrophil‐lymphocyte ratio

## Abstract

Inflammation plays a substantial role in COVID‐19 pathophysiology. Ferritin and neutrophil‐lymphocyte ratio (NLR) are significant prognostic biomarkers used in COVID‐19 patients, although they are affected by other factors such as comorbidities and age. Aging changes the immune system through immunosenescence and inflammaging; however, there are limited number of studies evaluating its effect on ferritin and NLR as part of the complete assessment for intensive care requirement and mortality risk. A single‐center retrospective cohort study of 295 COVID‐19 patients was performed at the Siloam Hospitals Makassar, South Sulawesi, Indonesia from April to August 2020. After admission, all patients were followed up for clinical outcomes. Patients were grouped into strata based on age (<50 years vs. ≥50 years) and risk groups (low‐risk ferritin vs. high‐risk ferritin; low‐risk NLR vs. high‐risk NLR). The endpoints of the study were the intensive care requirements and mortality. Among the 295 COVID‐19 patients, 264 survived and 31 deceased. Ferritin and NLR had higher area under curve (AUC) values than other inflammatory parameters and had significantly different outcomes in both mortality and intensive care requirement groups. The combination of ferritin and NLR showed higher AUC values for intensive care requirement and mortality (AUC, 0.783; 95% confidence interval, 0.703–0.864). Multivariate analysis showed that both endpoints were strongly affected by age, ferritin level, and NLR. Age significantly multiplied clinical endpoints in low‐risk group patients but not in high‐risk group patients. The combination of ferritin and NLR had a better predictive value for intensive care requirement and mortality risk. However, age strongly affects clinical outcome in low‐risk groups of both ferritin and NLR groups; hence, it should be considered as an early predictive factor of COVID‐19 disease progression.

## Graphical abstract

Combination of both ferritin and NLR had a better predictive value of intensive care requirement and mortality risk. However, age strongly affect clinical outcome in low‐risk groups (both ferritin and NLR groups), hence should be considered as early predictive factor of COVID‐19 disease progression.

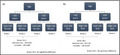

## 1 INTRODUCTION

Coronavirus is a large family of viruses that can cause diverse disease severity from common colds to serious illnesses such as the Middle East respiratory syndrome (MERS) and severe acute respiratory syndrome (SARS) (Cascella et al., 2020). In December 2019, a new coronavirus infection called coronavirus disease 2019 (COVID‐19) emerged. This infection is caused by a new virus the World Health Organization (WHO) named as severe acute respiratory syndrome coronavirus‐2 (SARS‐CoV‐2). Declared a global health emergency by the WHO (Kong and Agarwal, 2020), the COVID‐19 pandemic continues to weigh on country resources. It has been predicted that the rate of morbidity and mortality will continue to increase as COVID‐19 spreads in developing countries where the availability of health facilities is very limited (Lloyd‐Sherlock et al., 2020; Yang, Yu, et al., 2020). In Indonesia, the number of COVID‐19 cases is increasing rapidly due to the high transmission rate (Mohapatra et al., 2020), with the incidence fatality rate (IFR) varying by location (0.17%–1.7%) (Meyerowitz‐Katz & Merone, 2020). Indonesia also has a high fatality rate of approximately 8% in July 2020 and 3.9%–4% in September 2020 (WHO, 2019).

One of the biggest challenges in this pandemic was the lack of medical resources, especially the availability of intensive care unit (ICU) beds. Early identification of high‐risk patients for intensive care and mortality can improve COVID‐19 patient management and clinical outcomes in Indonesia. COVID‐19 patients are also classified based on disease severity: asymptomatic, mild, moderate, severe, and critically ill. Most patients infected with SARS‐CoV‐2 experience mild and moderate symptoms. On the other hand, severe patients tend to experience symptoms such as shortness of breath one week after infection. The symptoms of critically ill patients progressively evolve into acute respiratory failure, metabolic acidosis, coagulopathy, and septic shock (Meyerowitz‐Katz & Merone, 2020).

The hyperinflammatory response to SARS‐CoV‐2 infection plays an important role in disease severity and mortality (Del Valle et al., 2020). Inflammatory markers such as C‐reactive protein, procalcitonin, D‐dimer, neutrophil‐lymphocyte ratio (NLR), ferritin, inflammatory cytokines, and chemokines are elevated in COVID‐19 patients. The most significant predictors of inflammation in determining severity and mortality varied between studies (Velavan & Meyer, 2020). Ferritin and NLR are biomarkers used for disease severity and mortality assessment in many diseases and are recently reported as inflammatory markers for risk assessment in COVID‐19 patients (Imran et al., 2020; Li et al., 2020). The two are also affected by several factors such as age, sex, and comorbidities, which should be considered in the interpretation of ferritin levels and NLR.

COVID‐19 disease severity is associated with age (Perrotta et al., 2020). Aging causes major changes in immune response dysregulation, leading to a chronic systemic inflammatory state. Cytokines and chemokines also play a major role in the development of chronic inflammation and immunosenescence. However, there is a limited number of studies evaluating the effect of aging on ferritin and NLR as part of the complete assessment for intensive care requirement and mortality risk.

## 2 METHODS

### 2.1 Patients

This single‐center retrospective cohort study included 295 confirmed COVID‐19 patients and was performed at the Siloam Hospitals Makassar, South Sulawesi, Indonesia between April and August 2020. The inclusion criteria were as follows: (1) COVID‐19 confirmed by nasopharyngeal and oropharyngeal PCR swabs and (2) complete medical records. The exclusion criteria included the history of cancer, kidney failure, hypertension, and diabetes mellitus. Patients were treated in intensive care if any of the following is present: (1) respiratory distress with respiratory rates ≥30 times per min, (2) mean oxygen saturation room air ≤93%, (3) indication for mechanical ventilation, (4) shock, and (5) multiple organ failure.

All patients were evaluated for demographic data such as age, sex, location of care (ward or intensive care), clinical outcome (survived or deceased), laboratory parameters (white blood cell count, differential count, ferritin level, and NLR) at hospital admission. After admission, all patients were followed up for clinical outcomes. The endpoints of the study were intensive care admission and in‐hospital mortality.

### 2.2 Risk stratification

Based on the risk category, the low‐risk group included patients with low‐risk ferritin or low‐risk NLR, and the high‐risk group included patients with high‐risk ferritin or high‐risk NLR. Patients were further grouped into four strata based on age and groups of inflammatory biomarkers. The ferritin group included the following: strata 1 (<50 years, low‐risk ferritin); strata 2 (<50 years, high‐risk ferritin); strata 3 (≥50 years, low‐risk ferritin); and strata 4 (≥50 years, high‐risk ferritin). Meanwhile, the NLR group consisted of the following: strata 1 (<50 years, low‐risk NLR); strata 2 (<50 years, high‐risk NLR); strata 3 (≥50 years, low‐risk NLR); and strata 4 (≥50 years, high‐risk NLR).

### 2.3 Statistical analysis

Demographic data were also reported. Numeric variables, such as age, were reported as median (range), categorical variables were reported as percentages, and laboratory results were reported as mean (interquartile range). Bivariate analysis was performed using unpaired *t*‐test and chi‐square test. Statistical significance was set at *p* < 0.05. Ferritin, NLR, and age cut‐off were calculated based on the maximum Youden index (sensitivity + specificity − 1). Multivariate logistic regression analysis was performed on bivariate variables with *p* < 0.25 using the backward method to evaluate the strongest predictive factor for endpoint events. The null hypothesis was tested with the Hosmer–Lemeshow test to prove that there was no significant difference between the observed and expected values. All analyses were performed using SPSS version 22.0 for Windows (SPSS, Inc.).

## 3 RESULTS

The present study included 295 confirmed COVID‐19 cases. Among them, 264 survived and 31 deceased. Mean days until mortality was 6.5 ± 4.79 days (range, 1–22 days) after admission. Furthermore, the mean age of the patients was 47.4 ± 15.3 years (range, 20–90 years).

There was a significant difference between the groups’ endpoints for ferritin and NLR. Ferritin in survived and deceased groups were 1511.386 ± 2941.16 and 3264.58 ± 2941.1, respectively (95% confidence interval [CI], 532.37–2974.03; *p* = 0.005). Ferritin levels in the COVID‐19 isolation ward and ICU were 1425.94 ± 2806.8 and 3123.3 ± 4572, respectively (95% CI, 677.67–2717.1; *p* = 0.004). Meanwhile, the NLR value in survived and deceased groups were 4.78 ± 4.79 and 10.00 ± 8.4, respectively (95% CI, 3.25–7.1; *p* < 0.001). The NLR value in COVID‐19 isolation ward and ICU were 4.41 ± 4.34 and 9.53 ± 7.86, respectively (95% CI, 3.5–6.6; *p* < 0.001) (Table 1).

**TABLE 1:** Demographic data

	Total	Hospital care		*p* value	Mortality		*p* value
		COVID−19 isolation ward (*n* = 250)	Intensive care unit (*n* = 45)		Survived (*n* = 264)	Deceased (*n* = 31)	
Age (years)	47.4 ± 15.3	45.28 ± 15.09	56.78 ± 12.21	<0.001	46.24 ± 15.11	56.58 ± 13.83	<0.001
Sex							
Male	70.3%	84.3%	15.7%	0.497	88.2%	11.8%	0.136
Female	29.7%	80.7%	19.3%		93.2%	6.8%	
BMI index	21.95 ± 1.4	21.6 ± 1.5	22.5 ± 1.8	0.08	20.8 ± 1.9	23.5 ± 1.5	0.095
Laboratory value							
Leukocyte (10^3^/μl)	8.17 ± 4.31	7.45 ± 3.69	11.53 ± 5.33	<0.001	7.78 ± 4.08	11.38 ± 4.89	<0.001
Neutrophil (%)	67.8 ± 15.44	65.82 ± 15.35	77.46 ± 11.79	<0.001	66.72 ± 15.16	77.26 ± 14.89	<0.001
Lymphocyte (%)	20.86 ± 11.23	22.30 ± 10.96	14.18 ± 9.98	<0.001	21.82 ± 11.21	13.01 ± 7.90	<0.001
Monocyte (%)	8.55 ± 4.76	8.83 ± 4.95	7.1 ± 3.36	0.020	8.48 ± 3.93	9.01 ± 9.34	0.557
Thrombocyte (10^3^/μl)	240.96 ± 108.78	241.41 ± 103.22	238.16 ± 136.45	0.849	244.89 ± 105.07	206.87 ± 135.28	0.066
NLR	5.34 ± 5.50	4.41 ± 4.34	9.53 ± 7.86	<0.001 (3.5 −6.6)	4.78 ± 4.79	10.00 ± 8.4	<0.001 (3.25–7.1)
Ferritin (ng/ml)	1689.95 ± 3194.99	1425.94 ± 2806.8	3123.3 ± 4572	0.004 (677.67–2717.1)	1511.386 ± 2941.16	3264.58 ± 2941.1	0.005 (532.37–2974.03)

NLR, neutrophil‐lymphocyte ratio

### 3.1 Laboratory parameter for mortality risk and intensive care

The receiver operating characteristic (ROC) analysis of laboratory parameters on mortality risk showed an area under curve (AUC) value of 0.764 (95% CI, 0.672–0.857; *p* < 0.001), 0.703 (95% CI, 0.595–0.811; *p* < 0.001), 0.746 (95% CI, 0.652–0.844; *p* < 0.001), 0.743 (95% CI, 0.636–0.850, *p* < 0.001), 0.439 (95% CI, 0.309–0.569; *p* = 0.281), 0.389 (95% CI, 0.269–0.509; *p* = 0.051), and 0.222 (95% CI, 0.140–0.304; *p* < 0.001) for NLR, ferritin, neutrophil, leukocyte, monocyte, thrombocyte, and lymphocyte, respectively.

**FIGURE 1:**
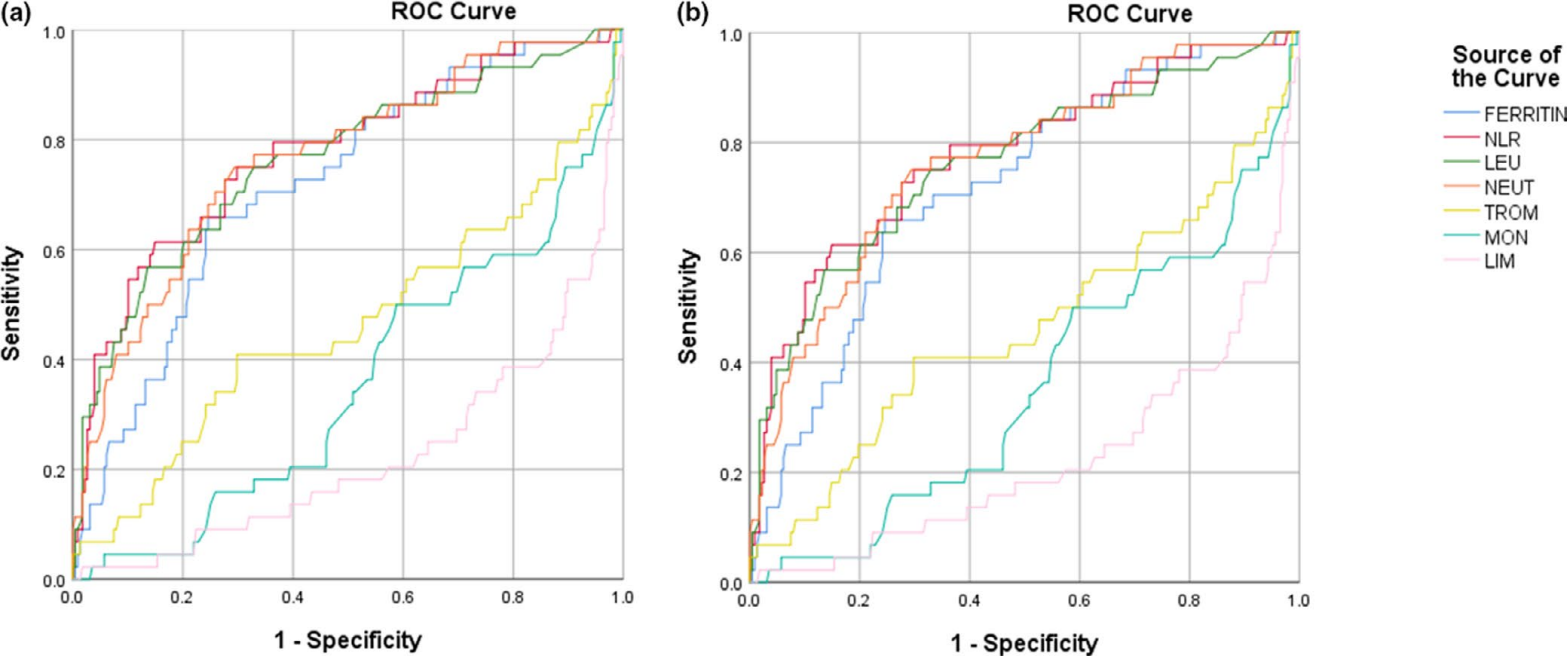
ROC analysis of laboratory parameters on (a) mortality risk and (b) intensive care requirement

The ROC analysis of laboratory parameters for intensive care requirement showed an AUC of 0.776 (95% CI, 0.695–0.858, *p* < 0.001), 0.719 (95% CI, 0.639–0.800; *p* < 0.001), 0.766 (95% CI, 0.686–0.845; *p* < 0.001), 0.762 (95% CI, 0.678–0.845; *p* < 0.001), 0.463 (95% CI, 0.358–0.568; *p* = 0.054), 0.355 (95% CI, 0.264–0.569; *p* = 0.445), and 0.225 (95% CI, 0.142–0.308; *p* < 0.001) (Figure 1) for NLR, ferritin, neutrophil, leukocyte, thrombocyte, monocyte, and lymphocyte, respectively.

**FIGURE 2:**
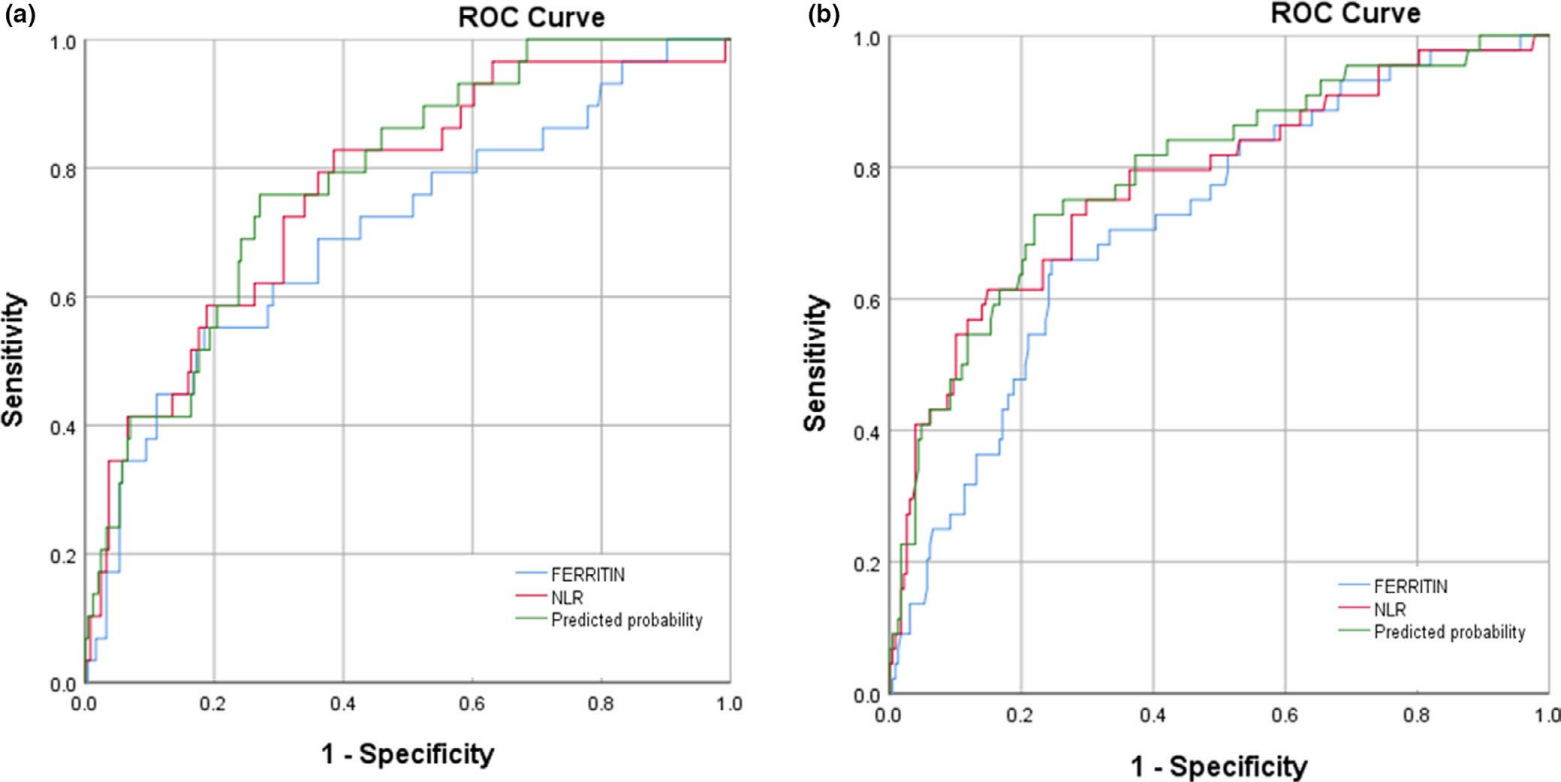
Analysis of ROC combination of ferritin and NLR for (a) mortality risk and (b) intensive care requirement

Ferritin and NLR were chosen for further analyses. The NLR was chosen over neutrophils and leukocytes due to its higher AUC. The ROC analysis of the ferritin and NLR combination showed an AUC of 0.783 (95% CI, 0.703–0.864) and 0.792 (95% CI, 0.716–0.868) for mortality risk and intensive care requirement, respectively (Figure 2).

After grouping according to cut‐off values, there was a significant difference between the ferritin and NLR values for intensive care requirement and mortality risk (*p* < 0.001). Ferritin value with a cut‐off of 1288.5 ng/ml had a sensitivity of 70.5% and specificity of 67% for intensive care requirement and a sensitivity of 69% and specificity of 63.7% for mortality risk. The NLR value with a cut‐off of 4.96 had a sensitivity of 79.5% and specificity of 63.5% for intensive care requirement and a sensitivity of 67.7% and specificity of 68.9% for mortality risk (Table 2).

**TABLE 2:** Cut‐off values of ferritin and NLR parameters for intensive care requirement and mortality risk

	Variable	Cut‐off	AUC (95% CI)	Sensitivity	Specificity	PPV	NPV	*p* value
Intensive care requirement	Ferritin	1288.5	0.719 (0.639–0.800)	70.5%	67%	78.8%	92.7%	<0.001
	NLR	4.96	0.776 (0.695–0.858)	79.5%	63.5%	81.6%	91.3%	0.03
Mortality risk	Ferritin	1288.5	0.703 (0.595–0.811)	69%	63.7%	78.9%	95.1%	0.001
	NLR	4.96	0.764 (0.672–0.857)	67.7	68.9	70.5%	94.9%	<0.001

### 3.2 Effect of age on ferritin and NLR values

**FIGURE 3:**
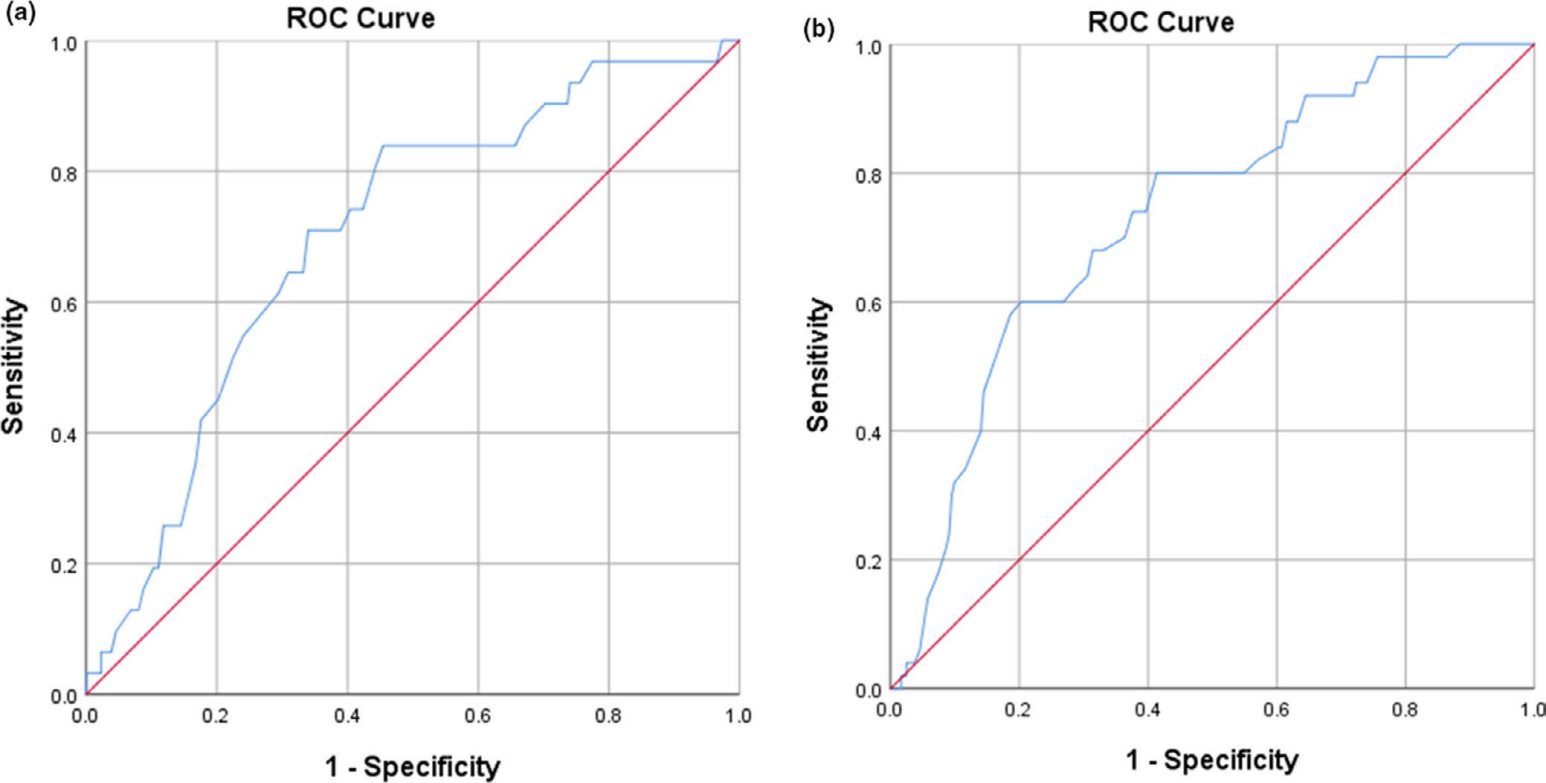
ROC analysis of age on (a) mortality risk and (b) intensive care requirement

This study showed an interrelationship between age and risk markers, with a significant difference between age and ferritin (*p* < 0.001; odds ratio [OR], 2.70; 95% CI, 1.635–4.439) and NLR (*p* = 0.37; OR, 1.711; 95% CI, 1.055–2.775) groups. Age on mortality risk and intensive care requirement also showed AUC values of 0.70 and 0.73, respectively. A cut‐off value of 50 years was determined, with sensitivity and specificity of 74.2% and 60%, respectively, for both factors (Figure 3).

Multivariate analysis showed that age (*p* = 0.04; OR, 2.59; 95% CI, 0.054–6.352), NLR (*p* < 0.001; OR, 5.13; 95% CI, 2.122–12.409), and ferritin (*p* = 0.005; OR, 3.58; 95% CI, 1.467–8.715) were predictors of mortality. Similar results showed that age (*p* = 0.002; OR, 3.53; 95% CI, 1.589–7.839), NLR (*p* < 0.001; OR, 6.5; 95% CI, 3.000–14.063), and ferritin (*p* < 0.001; OR, 4.29; 95% CI, 1.973–9.347) were predictors of intensive care requirement.

### 3.3 Age and risk stratification

In the ferritin group, strata 1 had 111 surviving patients, 2 deceased patients, 109 COVID‐19 isolation ward patients, and 4 ICU patients; strata 2 had 40 surviving patients, 6 deceased patients, 41 COVID‐19 isolation ward patients, and 5 ICU patients; strata 3 had 56 surviving patients, 8 deceased patients, 50 COVID‐19 isolation ward patients, and 14 ICU patients; and strata 4 had 57 surviving patients, 15 deceased patients, 50 COVID‐19 isolation ward patients, and 22 ICU patients (Table 3).

**TABLE 3:** Age and risk stratification

	Ferritin group				NLR group			
	Strata 1 (<50 years, low‐risk ferritin)	Strata 2 (<50 years, high‐risk ferritin);	Strata 3 (≥50 years, low‐risk ferritin)	Strata 4 (≥50 years, high‐risk ferritin)	Strata 1 (<50 years, low‐risk NLR)	Strata 2 (<50 years, high‐risk NLR)	Strata 3 (≥50 years, low‐risk NLR)	Strata 4 (≥50 years, high‐risk NLR)
Survived	111	40	56	57	110	41	69	44
Deceased	2	6	8	15	2	6	8	15
Isolation ward	109	41	50	50	108	38	60	44
ICU	4	5	14	22	4	9	17	15

* Number of patients in Ferritin and NLR Groups. There was a significant difference between strata 1 and 3, but there was no significant difference between strata 2 and 4 in both groups.

**FIGURE 4:**
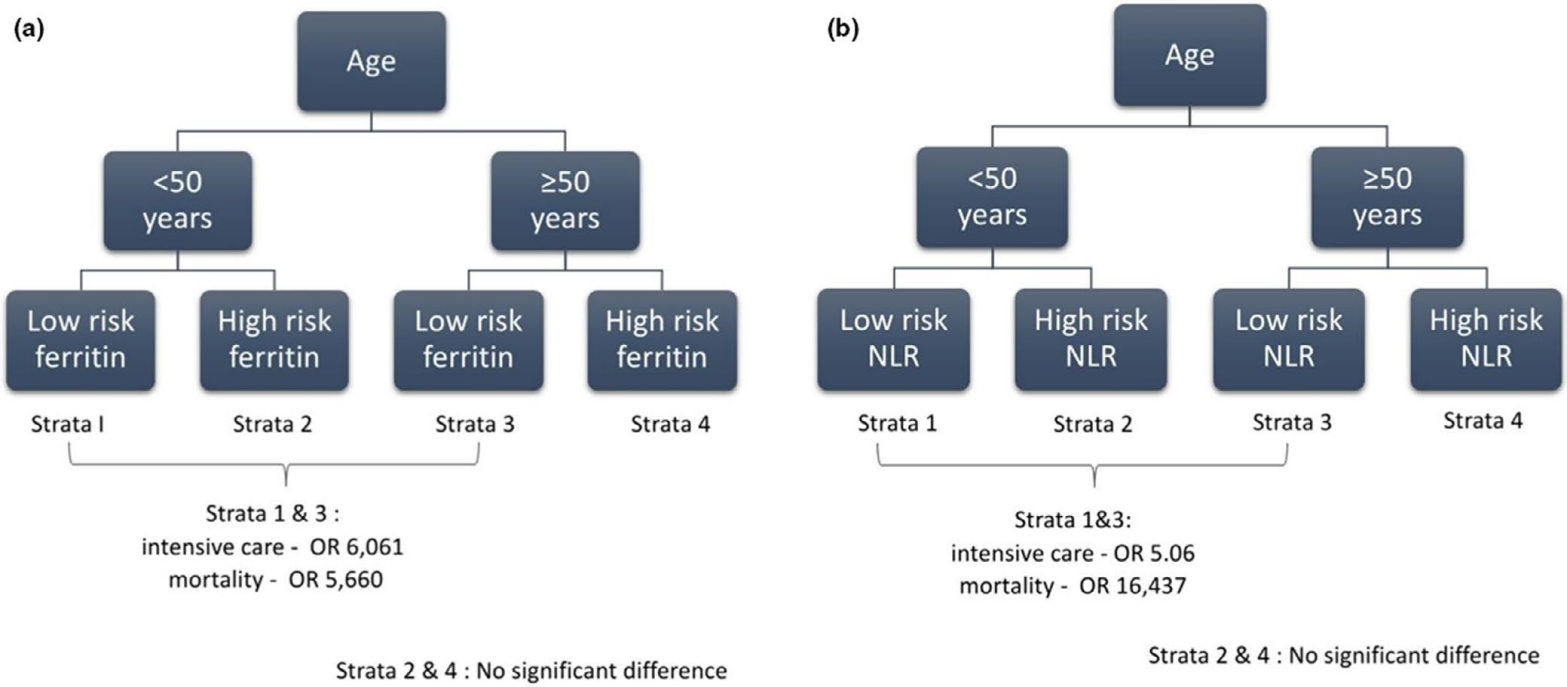
Risk stratification (a) ferritin group and (b) NLR group

Based on the mortality risk endpoint, there was a significant difference between strata 1 and 3 (*p* = 0.029; OR, 5.660; 95% CI, 1.104–29.02) and no significant difference between strata 2 and 4 (*p* = 0.174). Based on the intensive care requirement endpoint, there was a significant difference between strata 1 and 3 (*p* = 0.009; OR, 6.061; 95% CI, 1.570–23.398) and no significant difference between strata 2 and 4 (*p* = 0.082) (Figure 4).

In the NLR group, strata 1 had 110 surviving patients, 2 deceased patients, 108 COVID‐19 isolation ward patients, and 4 ICU patients; strata 2 had 41 surviving patients, 6 deceased patients, 38 COVID‐19 isolation ward patients, and 9 ICU patients; strata 3 had 69 surviving patients, 8 deceased patients, 60 COVID‐19 isolation ward patients and 17 ICU patients; and strata 4 had 44 surviving patients, 15 deceased patients, 44 COVID‐19 isolation ward patients, and 15 ICU patients (Table 3).

Based on the mortality risk endpoint, there was a significant difference between strata 1 and 3 (*p* < 0.001; OR, 16.437; 95% CI, 1.510–5.450) and no significant difference between strata 2 and 4 (*p* = 0.066). Based on the intensive care requirement endpoint, there was a significant difference between strata 1 and 3 (*p* < 0.001; OR, 5.061; 95% CI, 1.470–5.398) and no significant difference between strata 2 and 4 (*p* = 0.216) (Figure 4).

## 4 DISCUSSION

This study showed a strong correlation between ferritin and NLR on mortality risk and intensive care requirement compared with thrombocytes, monocytes, and lymphocytes. We reported that an NLR ≥4.96 and ferritin level ≥1288.5 ng/ml indicated a higher risk for intensive care requirement and mortality risk. We excluded several other comorbidities that may affect the NLR and ferritin levels to minimize bias.

The NLR is a simple and accessible inflammatory marker. Lymphopenia has been reported to be commonly observed within the first 2 weeks of SARS infection (He et al., 2005), with lymphocytes playing a pivotal role in SARS‐infected cell elimination (Deng et al., 2020). The underlying mechanisms of lymphocyte deficiency that contribute to NLR increase are the following: (1) SARS‐CoV‐2 directly infecting lymphocytes, and causing cell death; (Wang et al., 2020) (2) increased lactic acid in severe COVID‐19 patients inhibiting lymphocyte proliferation (T cell exhaustion and depletion progress in proportion to the inflammatory cytokines); and (3) SARS‐CoV‐2 directly invading and destroying lymphatic organs (spleen and lymph nodes), causing lymphopenia (Yang, Liu, et al., 2020).

Previous studies have shown that ferritin increases significantly in severe COVID‐19 patients (Vargas‐Vargas & Cortés‐Rojo, 2020). Ferritin is a key mediator in immune dysregulation, especially in extreme hyperferritinemia, through its immunosuppressive and pro‐inflammatory effects that contribute to cytokine storms (Abbaspour et al., 2014). Its ability to bind and store iron are associated with immune and inflammatory responses. SARS‐CoV‐2 interacts directly with the heme part of hemoglobin, resulting in increased heme levels along with Fe^2+^ ions. Iron causes destructive effect due to inflammation and ferroptosis, which triggers ferritin production to bind with free iron as a protective mechanism to reduce tissue damage (Dhont et al., 2020; Liu & Hualan, 2020).

Increased ferritin levels and NLR are generally known to be associated with patient outcomes. We added the novelty value from previous studies because we evaluated the relationship between ferritin and NLR for mortality risk and intensive care requirement in the age stratification perspective. Congruent with previous studies, independent ferritin and NLR were associated with mortality and severity in COVID‐19 patients. We also found that the NLR and ferritin combination can increase the predictive value of mortality risk and intensive care requirement.

Interestingly, our study found a significant change in the mortality risk and intensive care requirement of patients aged ≥50 years in low‐risk ferritin and NLR groups (strata 1 vs. strata 3) but not in high‐risk ferritin and NLR groups (strata 2 vs. strata 4). This suggests that age significantly affected endpoints in the low‐risk groups. Whether age is an independent risk for or if comorbidities play a role with increasing age in COVID‐19 mortality and severity is still debatable (Romero Starke et al., 2020). Ho et al. reported comorbidities increased mortality risk among older adults, with age as an independent risk factor. The risk stratification model can prove this hypothesis. Nonetheless, age had an independent role in the low‐risk ferritin and NLR groups (Ho et al., 2020).

Ferritin and NLR risk stratification based on age group can help clinicians during pandemics when the health care workers and facilities are scarce. Risk stratification can help organize medical care, especially in high‐risk critically ill patients. Patients aged <50 years with NLR <4.96 or ferritin <1288.5 ng/ml (strata 1) had a lower risk for intensive care requirement and mortality risk; hence, they can be treated in general COVID‐19 wards or home isolation. Meanwhile, patients aged ≥50 years with NLR <4.96 or ferritin <1288.5 ng/ml (strata 3) had multiple risks for intensive care requirement (5.06–6.06 times) and mortality (5.66–16.43 times). This can be a predictive factor for clinicians, despite the patient's low‐grade inflammatory marker profile.

The underlying hypothesis behind this effect is immune system aging. Although changes in lymphocyte count and its subpopulation are markers of immunosenescence (Aiello et al., 2019), they cannot fully explain this relationship. Individual characteristics are some of the independent factors because immune system aging varies between individuals (Lin et al., 2016; Montecino‐rodriguez et al., 2013). One of the prominent signs of immune system aging is naive lymphocyte reduction due to thymus involution, hematopoietic stem cells (Sadighi Akha, 2018), and inadequate peripheral maintenance (Lin et al., 2016). Other factors include inflammaging, which is a low‐grade, sterile, and systemic inflammation that reflects intercellular signaling disruption and acts as a sign of integrative aging (Sadighi Akha, 2018).

The effect of aging on ferritin levels is not completely understood, with several studies reporting contradicting results. Serum ferritin levels at 65–70 years old were reported to be twice higher than in patients aged 45–50 years (Ma et al., 2016). In addition, ferritin increasing with age is a sign of inflammaging (Cankurtaran et al., 2012), and there is a complex feedback between ferritin and cytokine in controlling pro‐ and anti‐inflammatory mediators (Kell & Pretorius, 2014; Rosário et al., 2013). Iron status is also affected by inflammation. Active ferritin production by macrophages and cytokines causes hyperferritinemia, resulting in the increase of several proinflammatory (IL‐1) and anti‐inflammatory (IL‐10) cytokines (Cankurtaran et al., 2012). Ferritin synthesis is also induced by inflammatory stimuli such as cytokine IL‐6 (Rosário et al., 2013), which is crucial in the COVID‐19 pathogenesis and increases in an age‐dependent manner (Puzianowska‐Kuźnicka et al., 2016). Furthermore, a few studies were evaluating ferritin in COVID‐19. Wu et al. (2020) and Zhou et al. (2020) reported that ferritin levels >300 ng/ml were associated with respiratory failure and mortality.

Our data from South Sulawesi, one of the largest islands in Indonesia, showed a mean ferritin level of 1689 ng/ml, which was higher than the eight previous studies with a range of 155.7–500 ng/ml. The mean NLR in our study was also higher (5.34) than that of 20 studies in the meta‐analysis (2.80). These data provide a brief description of COVID‐19 severity in Indonesia.

A limitation of our study was the broad division of age groups into two categories (<50 years and ≥50 years). More detailed age groups may be able to evaluate more specifically the effect of aging on ferritin and NLR for endpoints. Additionally, we had a small sample size and a retrospective view. A larger study with complete comorbidity evaluation is necessary for future evaluation.

## 5 CONCLUSION

Ferritin and NLR levels can be used as prognostic biomarkers to predict intensive care requirements and mortality risk. The combination of both parameters had a better predictive value. Patients with low‐risk ferritin and low‐risk NLR, but with different age categories, had significantly different risk outcomes. Thus, age should be considered as an early predictive factor of COVID‐19 disease progression.

## CONFLICT OF INTEREST

The authors have no conflicts of interest to declare.

## AUTHOR CONTRIBUTIONS

Rasyid H: Conceptualization, Methodology, Formal Analysis, Ethics, Recruiting, Investigation, Data collection, Supervision Writing‐Manuscript & Editing. Sangkereng A: Conceptualization, Methodology, Formal Analysis, Ethics, Recruiting, Investigation, Data collection, Writing‐Manuscript & Editing. Harjianti T: Conceptualization, Methodology, Formal Analysis, Investigation, Data collection, Writing‐Manuscript & Editing. Soetjipto AS: Conceptualization, Methodology, Formal Analysis, Ethics, Investigation, Data collection, Writing‐Original Draft.

## ETHICAL APPROVAL AND CONSENT TO PARTICIPATE

The study was approved by Ethical committee of Siloam Hospitals Makassar (No.224/SHMK‐DIR/XI/2020).

## Data Availability

The data are available from the corresponding author on reasonable request.
